# Editorial: Traumatic brain injury and post-traumatic stress disorder: from neurobiology to treatment

**DOI:** 10.3389/fpsyt.2025.1560177

**Published:** 2025-03-28

**Authors:** James P. Kelly, Jeffrey D. Lewine, Kaloyan S. Tanev

**Affiliations:** ^1^ Department of Neurology, School of Medicine, University of Colorado, Aurora, CO, United States; ^2^ Department of Psychology, University of New Mexico, Albuquerque, MN, United States; ^3^ Mind Research Network (MRN), Albuquerque, MN, United States; ^4^ Department of Psychiatry, Massachusetts General Hospital, Harvard Medical School, Boston, MA, United States; ^5^ Home Base Clinic, Boston, MA, United States

**Keywords:** PTSD - posttraumatic stress disorder, TBI - traumatic brain injury, CBT (cognitive behavioral therapy), massed treatment, invisible

Both traumatic brain injury (TBI) and posttraumatic stress disorder (PTSD) are highly prevalent in the USA and internationally. In addition to their high prevalence among civilians, they have become known as the signature, but invisible, wounds of war among service members and veterans. The yearly incidence of TBI in the USA had been estimated to be about 1.7 million (1). However, a more recent review of 2009-2010 TBI data by the National Academies of Sciences, Engineering, and Medicine found that 4.8 million U.S. citizens were evaluated in emergency departments for TBI each year (2). Although the majority of new TBIs are mild, and most people with mild TBI (mTBI) recover fully within 3-12 months, a significant minority go on to develop persistent post-concussive symptoms. Disability from TBI is generally proportionate to the severity of TBI, but even those who suffer an only “mild” TBI may face partly debilitating long-term cognitive, emotional-behavioral, vocational, and social reintegration consequences (3–5), with higher rates reported among service members. According to the National Center for PTSD, 14% of men and 24% of women who accessed VA healthcare in 2024 carried diagnoses of PTSD^1^. In considering the inter-relationships between TBI and PTSD it should be noted that PTSD often develops in the absence of TBI, but the presence of a TBI more than doubles the risk for the development of PTSD, presumably because of a degree of shared neurobiology.

TBI and PTSD share comorbidities such as depression, anxiety, substance use, cognitive dysfunction, and difficulty reintegrating. When a single event is responsible for both TBI and the subsequent development of PTSD, distinguishing between the neurological and psychological etiology of specific symptoms is often difficult, if not impossible. Diagnosis relies on combination of symptoms and signs. Recently, a number of promising biomarkers have been researched for diagnosis and treatment follow up of TBI and PTSD, but these have not yet been integrated in common clinical use.

There are only a few evidence-based treatments for amelioration of the symptoms of TBI and PTSD. Cognitive rehabilitation is often used to treat the consequences of TBI. This has some established efficacy when applied immediately after, or even years after, injury (6, 7). Symptom-based pharmacotherapies may also be helpful, but the presence of brain injury often demands modification of traditional dosing strategies. Evidence-based treatments for PTSD include several types of trauma-focused psychotherapies and medications. For TBI and PTSD resulting from the same event, there are currently no established standards of treatment, although intensive outpatient treatment programs in the military and veteran systems of care have shown benefit as measured by widely accepted clinical outcome metrics.

In the last two decades, there has been increasing evidence for neuropsychiatric consequences of TBI. TBI is associated with high prevalence of depression, irritability, and cognitive dysfunction. Selective serotonin reuptake inhibitors (SSRIs) have been shown to be effective in improving depressive symptoms after TBI, although they have not differentiated from placebo. SSRIs have also been shown to mitigate against depressive symptoms after TBI when given preventatively. Stimulants have been shown to improve cognition and behaviors after TBI, and cholinesterase inhibitors have been shown effective for memory problems in those with severe memory problems. For PTSD, cognitive behavioral therapies (CBTs) have shown to be effective in 50-60% of patients who complete therapy, but CBTs such as prolonged exposure suffer from high dropout rates. Additionally, there is a lack of adequate numbers of qualified mental health providers for administration of CBT. To mitigate some of these delivery problems, massed delivery CBTs have been developed over the past decade. These allow the administration of CBTs in a condensed, sometimes daily, format. These mass approaches have gained popularity and have been shown to be effective. Involvement of the patient’s family is essential to treatment of both TBI and PTSD, because the patient affects the family system, and the family plays a crucial role in the patient’s readaptation into society.

Research using resting-state functional magnetic resonance imaging (rs-fMRI), magnetoencephalography (MEG), and quantitative electroencephalography (qEEG) has implicated a number of specific brain circuits in TBI and PTSD, with novel neuromodulatory treatments showing promise in ameliorating the dysfunction in these circuits and thereby improving symptoms. Emerging treatments include transcranial magnetic stimulation (TMS), transcranial direct current stimulation (tDCS), vagal nerve stimulation (VNS), and photobiomodulation.

There is a clear need to further develop clinical practice guidelines (CPGs) for the diagnosis and treatment of TBI and PTSD. These CPGs should address diagnosis (both high and low-tech), triage (primary care, specialists), support of TBI and PTSD survivors in different phases after neurological or psychological injury (i.e., acute, post-acute, and chronic), as well as diagnosis and treatment of TBI and PTSD resulting from the same event(s). These CPGs need to be adapted for both high-income as well as low-income countries, with decision-tree analyses taking into account regional resource availability.

The articles in this Research Topic highlight different aspects of the above issues. One of the articles (Buddenbaum et al.) presents the associations between repetitive head impact and mental health problems among former amateur athletes, decades after these impacts were sustained. It highlights the neuropsychiatric and neurobehavioral consequences of TBI, the association between TBI and PTSD, and the influence of socioeconomic status on mental health symptoms. A second article (Harward et al.) presents an innovative delivery model for veterans and service members with TBI and PTSD comprising massed CBTs and other integrative treatments. A third article (Hoover et al.) highlights the importance of involving families in patients’ treatment and of treating comorbid substance use disorders. A fourth article (Adugna et al.) presents the rate of PTSD and associated factors among military service personnel admitted to a military hospital in Eastern Ethiopia. A fifth article (Bailar-Heath et al.) presents a retrospective chart review of TMS for PTSD and depression in active-duty special operations service members. All in all, these articles present different aspects of TBI, PTSD, associated neuropsychiatric symptoms, demographic and environmental factors, as well as innovative models for treating these conditions.

Diagnostic and therapeutic strategies for TBI and PTSD remain under active development, and as exemplified by the articles in this volume, viable solutions are forthcoming.

## Footnotes

1
www.ptsd.va.gov/understand/common/common_veterans.asp.

## References

[1] Faul M XU L Wald MM Coronado VG. Traumatic brain injury in the United States: emergency department visits, hospitalizations and deaths 2002–2006. Centers Dis Control Prev. (2010).

[2] Berwick D Bowman K Matney C eds. A consensus study report of the National Academies of Sciences, Engineering and Medicine. Washington, DC: The National Academies of Sciences, Engineering and Medicine (NASEM) (2022). 10.1001/jama.2022.0089 35103760

[3] Vitaz TW Jenks J Raque GH Shields CB. Outcome following moderate traumatic brain injury. Surg Neurol. (2003) 60:285–91; discussion 291. doi: 10.1016/s0090-3019(03)00378-1 14505837

[4] Ponsford JL Downing MG Olver J Ponsford M Acher R Carty M. Longitudinal follow-up of patients with traumatic brain injury: outcome at two, five, and ten years post-injury. J Neurotrauma. (2014) 31:64–77. doi: 10.1089/neu.2013.2997 23889321

[5] Kilpatrick DG Resnick HS Milanak ME Miller MW Keyes KM Friedman MJ. National estimates of exposure to traumatic events and PTSD prevalence using DSM-IV and DSM-5 criteria. J Trauma Stress. (2013) 26:537–47. doi: 10.1002/jts.21848 PMC4096796 24151000

[6] Cicerone KD Langenbahn DM Braden C Malec JF Kalmar K Fraas M. Evidence-based cognitive rehabilitation: updated review of the literature from 2003 through 2008. Arch Phys Med Rehabil. (2011) 92:519–30. doi: 10.1016/j.apmr.2010.11.015 21440699

[7] Cicerone KD Goldin Y Ganci K Rosenbaum A Wethe JV Langenbahn DM. Evidence-based cognitive rehabilitation: systematic review of the literature from 2009 through 2014. Arch Phys Med Rehabil. (2019) 100:1515–33. doi: 10.1016/j.apmr.2019.02.011 30926291

